# A Computationally Designed Water-Soluble Variant of a G-Protein-Coupled Receptor: The Human Mu Opioid Receptor

**DOI:** 10.1371/journal.pone.0066009

**Published:** 2013-06-14

**Authors:** Jose Manuel Perez-Aguilar, Jin Xi, Felipe Matsunaga, Xu Cui, Bernard Selling, Jeffery G. Saven, Renyu Liu

**Affiliations:** 1 Department of Chemistry, University of Pennsylvania, Philadelphia, Pennsylvania, United States of America; 2 Department of Anesthesiology and Critical Care, University of Pennsylvania, Philadelphia, Pennsylvania, United States of America; 3 Department of Anesthesiology, Beijing Tongren Hospital, Capital Medical University, Beijing, China; 4 Impact Biologicals Inc., Swarthmore, Pennsylvania, United States of America; University of Michigan, United States of America

## Abstract

G-protein-coupled receptors (GPCRs) play essential roles in various physiological processes, and are widely targeted by pharmaceutical drugs. Despite their importance, studying GPCRs has been problematic due to difficulties in isolating large quantities of these membrane proteins in forms that retain their ligand binding capabilities. Creating water-soluble variants of GPCRs by mutating the exterior, transmembrane residues provides a potential method to overcome these difficulties. Here we present the first study involving the computational design, expression and characterization of water-soluble variant of a human GPCR, the human mu opioid receptor (MUR), which is involved in pain and addiction. An atomistic structure of the transmembrane domain was built using comparative (homology) modeling and known GPCR structures. This structure was highly similar to the subsequently determined structure of the murine receptor and was used to computationally design 53 mutations of exterior residues in the transmembrane region, yielding a variant intended to be soluble in aqueous media. The designed variant expressed in high yield in *Escherichia coli* and was water soluble. The variant shared structural and functionally related features with the native human MUR, including helical secondary structure and comparable affinity for the antagonist naltrexone (*K*_d_  = 65 nM). The roles of cholesterol and disulfide bonds on the stability of the receptor variant were also investigated. This study exemplifies the potential of the computational approach to produce water-soluble variants of GPCRs amenable for structural and functionally related characterization in aqueous solution.

## Introduction

The G-protein-coupled receptor (GPCR) family of proteins have important roles in signal transduction and cellular response to extracellular stimuli [1] and are the targets of many pharmaceuticals. Drug development and the study of the molecular mechanisms of (GPCRs) are impeded by limited solubility and difficulty in isolating sufficient quantities of functional receptors. These difficulties are caused in part by the large numbers of hydrophobic residues on the transmembrane, lipid-contacting protein exterior. To circumvent these problems, water-soluble variants of GPCRs can potentially be identified by systematically redesigning these exterior residues. Along these lines, computational protein design has been used to create water-soluble analogs of transmembrane proteins that can be expressed in *E. coli* and that retain structural and functional features of their parent membrane proteins [2], [3], e.g., the bacterial potassium channel (KcsA) [4] and a transmembrane domain of the nicotinic acetylcholine receptor (nAChR) [5]. Herein, such design is extended to a member of the GPCR superfamily, where comparative modeling is used to identify exterior residues in the transmembrane region.

The mu opioid receptor (MUR) is a GPCR that is the dominant target of opioids, many of which are potent analgesics widely used for the treatment of severe and chronic pain, e.g., morphine [6]. Opioid use has soared in recent years [7]–[9], and human MUR has been linked to many of its notorious side effects, including addiction and deadly respiratory depression [6], [7]. The molecular mechanisms governing GPCR function remain obscure despite the profound insights obtained recently from multiple high-resolution crystal structures [10]–[18].

Here computational redesign to increase water solubility while retaining functionally related properties was applied to the human MUR. In previous redesign efforts, template structures were derived from experimental structures of KcsA (via X-ray diffraction) [4], [19] and nAChR (via cryo-electron microscopy) [5], [20]. Often with membrane proteins (including GPCRs), such experimentally determined structures are not available. No structure for the human MUR was available when this study was initiated, thus the approach was extended to include structural modeling. The design involved several key steps: (*i*) Comparative modeling using sequence alignment and known GPCR structures (the subsequently solved structure of murine MUR provided a means to assess the quality of the comparative model [15]); (*ii*) Identification and co mputational redesign of transmembrane exterior residues; (*iii*) Overexpression in *E. coli* and purification; (*iv*) Characterization of structural and ligand-binding properties in aqueous buffer. The designed water-soluble human MUR has structurally and functionally related properties comparable to the native membrane-soluble human MUR.

## Materials and Methods

### Comparative Modeling

Bovine rhodopsin (UniProtKB accession number P35372; PDB accession code: 1U19) [11] and the β_2_ adrenergic receptor (UniProtKB: P02699; PDB: 2RH1) [12] were used as templates in the creation of models of the human MUR transmembrane domain (UniProtKB: P07550) [21]. Pairwise sequence alignments of the human mu opioid receptor with bovine rhodopsin and with the β_2_ adrenergic receptor were carried out using BLASTp [22] with the Blosum62 substitution matrix [23]. In a multiple sequence alignment, adjustments were performed to maintain highly conserved residues of the class A GPCR family [24]. One hundred independent models of a protein structure comprising residues S66 to C353 were generated using Modeller 8v2 [25]. The structure was validated using molprobity [26] and then validated a posteriori by the recently solved crystal structure of the murine mu opioid receptor [15].

### Computational Protein Design

Within the comparative model structure, residues targeted for mutation were those having more than 40% solvent exposure (1.4 Å probe radius and percent exposure measured relative to GXG tripeptide) [27] and were within previously estimated membrane boundaries [28]. To identify the site-specific amino acid probabilities of the target positions, a statistical entropy-based formalism was used [4], [29], [30]. In this theoretical approach, an effective entropy, which was a function of the site-specific probabilities of the amino acids and their conformational states, was maximized by varying the probabilities subject to energetic constraints on the sequences using a Lagrange multiplier method. The set of probabilities corresponding to the optimum was used to guide protein redesign. Energy functions to quantify sequence-structure compatibility were derived from a molecular mechanics force field [31]. To account for solvation effects and for the tendency of different amino acids to be exposed to or sequestered from water (hydrophobicity), an effective energy (herein environmental energy) was employed that was based on the local density of C_β_ atoms of each residue and parameterized using a database of soluble, globular proteins [4], [30]. In this case the environmental energy term was constrained to a value expected for soluble proteins having 288 residues [4], [30], the size of the TM domain of the human MUR [4]. The conformational variability of the amino acid residues was addressed using a rotamer library of side chain conformations [32]. The site-specific probabilities of the amino acids at each of the variable positions were determined by maximizing an effective entropy function subject to constraints on the two energies. These probabilities were used to identify specific sequences. After the residues targeted for potential mutations were identified, the remaining residues were fixed at their wild type identities, and their side chain conformations were allowed to vary to accommodate possible mutations. All amino acids but proline and cysteine were permitted at each of the identified variable positions. Calculations proceeded as described previously [4]. Probabilities used were those for which the Lagrange multiplier β conjugate to the average molecular force field energy took on a value of β^−1^ = 0.5 kcal/mol. Identification of sequence proceeded iteratively until amino acid identities were specified at each of the targeted residues.

### Protein Expression and Purification

The synthetic cDNA encoding of the transmembrane-only water-soluble MUR variant (wsMUR-TM) was produced by DNA2.0 Inc. (Menlo Park, CA). The sequences were subcloned between the *Nde*I and *Xho*I restriction sites of the expression plasmid pET-28b(+) (EMD/Novagen). *E. coli* BL21(DE3) cells (EMD/Novagen) were used for expression. Cells were grown in shake flasks with Lysogeny broth medium with 30 µg/mL kanamycin to an OD of 1.0, induced with 1 mM Isopropyl β-D-1-thiogalactopyranoside (IPTG) for 3 h at 37°C, then pelleted by centrifugation. Cell pellets were stored at 20°C until purification. For solubility testing, 1 OD aliquots of cells were pelleted in microcentrifuge tubes, suspended in 150 µL of TE (50 mM Tris-HCl, 1 mM EDTA, pH = 8.0), then shaken with 0.3 g of glass beads (0.1 mm diameter) for 5 min. Aliquots of the resulting lysates were spun in a microcentrifuge for 1 min. Aliquots of total lysate, or the supernatant and pellet fractions after centrifugation, were analyzed on reducing sodium dodecyl sulfate (SDS) gels.

Frozen cells from 250 mL of fermentation (500–550 ODs) were thawed, and then suspended in 33.5 mL of 50 mM Tris-HCl, 1 M urea, pH = 8.0. Once the pellet was fully resuspended, EDTA was added to 1 mM, Triton X-100 to 1%, and hen egg lysozyme to 1 µg per OD of cells, in a total volume of 37 mL. After the slurry was incubated for 20 min at room temperature (RT), MgCl_2_ was added to 3 mM, followed by 100 units of benzonase. The suspension was swirled, incubated another 5 min at RT, and then spun in an Oak Ridge tube at 10,000 rpm for 20 min at 20°C in an SS-34 rotor (r_avg_  = 6.98 cm, r_max_  = 10.70 cm).

The resulting pellet was resuspended into 35 mL of 50 mM Tris-HCl, 1 M urea, pH = 8.0. Triton X-100 (1.5 mL of a 25% solution) and 2-mercaptoethanol (2-ME) was added to 40 mM. The tube was inverted several times, and then spun as above.

The following steps were designed to resemble those that had been used to dissolve and purify recombinant forms of native mu opioid receptor. The pellet from the above washes was resuspended into 5 mL of buffer phosphate Tris buffer (100 mM phosphate, 10 mM Tris, adjusted to pH = 8.0 with NaOH) and dispersed by drawing through a pipet followed by a 25 gauge needle. The volume was then raised to 37 mL by addition of phosphate Tris buffer, and 2-ME was then added to 40 mM. The tube was inverted to mix, then spun as above.

The resulting pellet was dispersed into 36 mL of PT as described above. The suspension was then mixed with an equal volume of phosphate Tris buffer containing 0.2% SDS and 10 mM 2-ME. The suspension was rocked until it became almost clear (60–90 min). The suspension was then poured into two 38 mL Oak Ridge tubes. These were spun tube at 12,000 rpm for 20 mins at 20°C in an SS-34 rotor.

### Mass Spectrometry

The appropriate protein band from an SDS-PAGE gel was excised and digested with trypsin. Peptides were injected into a nano-LC/MS (10 cm C18 capillary column) to be separated by Eksigent. NanoLC proteomics experiments were run at 200 nL/min for 60 min with gradient elution. Nanospray was used to spray the separated peptides into LTQ (Thermo Fisher Scientific, MA). The raw data was acquired by Xcalibur (Xcalibur, Inc. Arlington). Sequest (http://fields.scripps.edu/sequest/) was used to search the database Uniprot_Sprot, Scaffold 2.6 (Proteome Software, Inc. OR) was used to combine and analyze the Sequest generated data quantitatively by using spectrum count.

### Circular Dichroism and Thermal Stability

Circular dichroism (CD) spectra were recorded by using CD Spectrometer (Chirascan, AppliedPhotophysics Limited, Leatherhead, United Kingdom) with a scan speed of 1 nm/s and 1 mm path length. Corresponding blanks were used for calibration for each assay and subtracted from raw data. Two data sets were recorded and averaged to increase the signal-to-noise ratio. The CDNN CD spectra deconvolution software [33] was utilized to determine the secondary structure content of the proteins. CD spectroscopy for wsMUR-TM at different temperatures were recorded with 6 µM of the receptor in buffer (5 mM sodium phosphate, pH = 7.0) from 10°C to 90°C in increments of 2°C per min. Absorbance was maintained lower than 1.0 to ensure sufficient light transmission. The temperature-dependence curve was plotted using GraphPad Prism (version 5, GraphPad Software, Inc. La Jolla).

### Protein Unfolding and Thermal Stability

The CD spectra of the protein was determined in the presence and absence of 8 M urea with and without 2-ME (25 mM or 200 mM, 5 mM sodium phosphate, 0.01% SDS, pH = 7.0). The final samples contained protein diluted to 6 µM and the requisite dilutions of urea and 2-ME. Each sample was incubated at room temperature for 1 h.

CD spectroscopy was utilized to investigate the thermostabilization of wsMUR-TM by cholesterol. The CD spectra were recorded with a 6 µM of the receptor (0.27 mg/ml) in buffer (5 mM sodium phosphate, 0.01% SDS, pH = 7.0) from 10°C to 90°C. 0.01% SDS was used since cholesterol can be dissolved in SDS solution. Cholesterol with 1∶1 molar ratio to the protein was used. The thermostability determination protocol is similar as described above.

Protein unfolding was also studied by monitoring the intrinsic tryptophan fluorescence of the protein. A RF-5301PC spectrofluorophotomer (Shimadzu North America, Columbia, MD) was used to monitor fluorescence emission following excitation at 295 nm. Samples were prepared as in CD unfolding experiments. Samples were measured in duplicate and results reflect averaged values of each trial.

### Homogeneous Time-Resolved Fluorescence (HTRF) Based Binding Assay

The fluorescent binding assay employs the native MUR fused at the N-terminus to a SNAP-tag® enzyme and expressed on HEK293 cells. SNAP-tag-mu-opioid is then covalently labeled with terbium cryptate (Lumi4®-Tb), a long lifetime FRET donor. An analog of the potent opioid antagonist naltrexone that contains the d2 dye (red-naltrexone) is used as the fluorescence energy transfer acceptor. Upon ligand binding, a FRET process occurs between the Lumi4-Tb donor (emission at 620 nm) in SNAP-Lumi4-Tb-mu-opioid receptor and the red-naltrexone acceptor (emission at 665 nm). The fluorescence emission from the acceptor is detected in a time resolved manner (TR-FRET). For HTRF assay (Cisbio Bioassays, Bedford, MA), Tag-lite**®** mu opioid cells suspended in culture medium were dispensed in white 384-well low-volume microplates (Greiner Bio-one Greiner Bio-One North America, Monroe, NC) at 3700 cells/10 µL/well Tag-lite m opioid labeled cells with 60 nM of Tag-lite opioid receptors red-naltrexone, and 5 µL of the wsMUR-TM with 11 further 1∶2 serial dilutions from the µM to the nM range. All samples were mixed with final volume 20 µl and incubated at RT for 2 h. After incubation, HTRF signals were measured using a plate reader (BMG, Cary, NC) after excitation at 337 nm at both 620 and 665 nm emission, HTRF signal was calculated as a two-wavelength signal ratio: [intensity (665 nm)/intensity (620 nm)]. IC_50_ determination and statistical analysis IC_50_ values for wsMUR-TM were determined by fitting the dose–responses curves using the Prism program (GraphPad Software, San Diego, CA).

## Results

### Computational Design of a Water-Soluble Variant of the Human MUR

**Figure 1:**
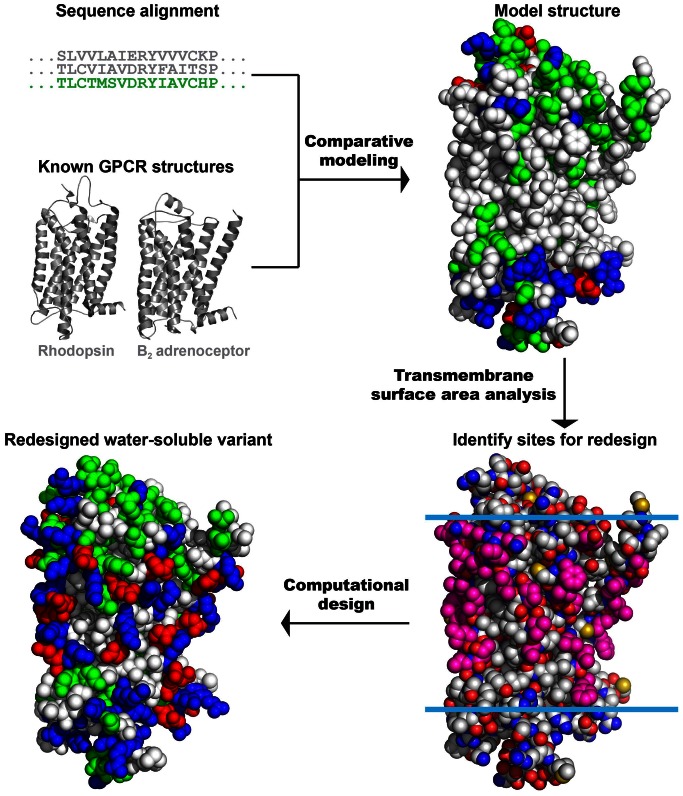
Scheme of the computational design protocol. *Step 1, Comparative modeling*: Starting from the sequence alignment between known GPCR structures (bovine rhodopsin and β_2_ adrenergic receptor) and human MUR, a model structure of the human MUR was generated. *Step 2, Identification of exposed sites in the transmembrane portion*: Using the comparative model, the transmembrane lipid-exposed positions were identified (pink). *Step 3, Computational design of selected exterior positions to generate a water-soluble variant*: The selected exterior positions are targeted of the computational redesign with the intention of increasing the protein’s solubility and ability to be overexpressed in *E. coli*. Residues are colored by amino acid types: hydrophilic in green (GNQSTY); hydrophobic in white (ACFILMPVW); basic in blue (HKR); and acidic in red (DE).

**Figure 2:**
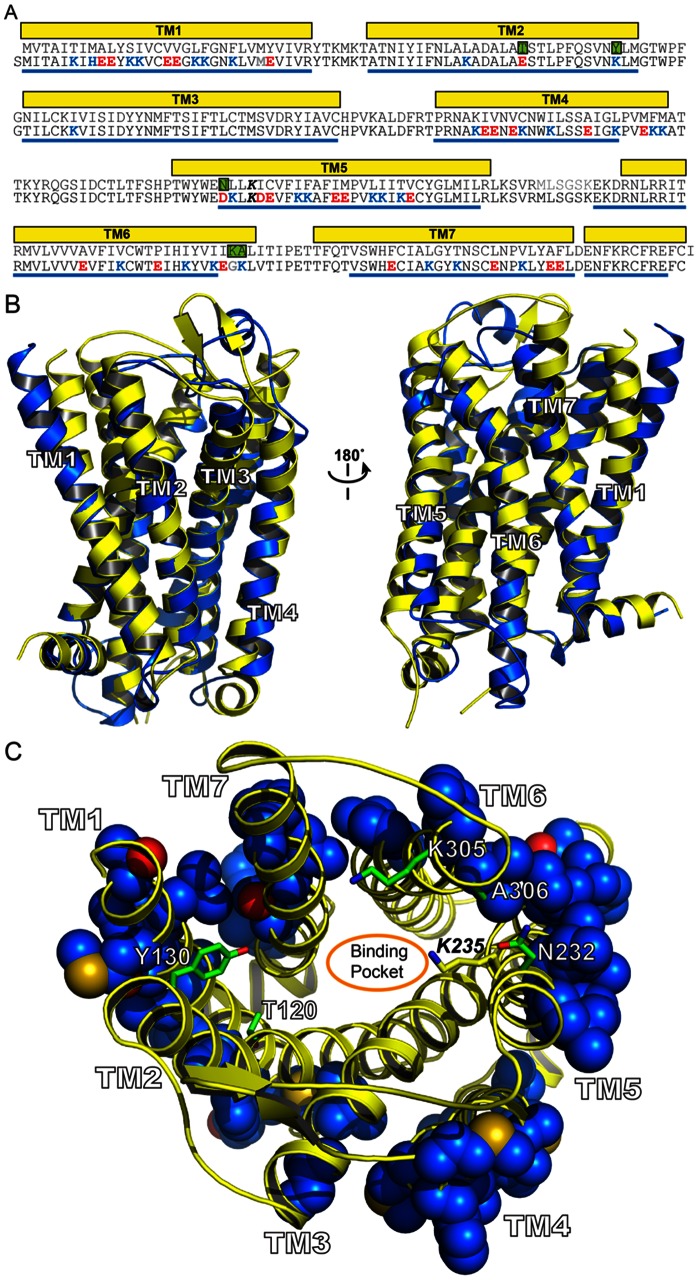
(A) Sequences of the crystal structure of the mouse mu opioid receptor (PDB code 4DKL; top) (1) and the human water-soluble variant wsMUR-TM (bottom). The murine sequence (top) corresponds to that whose structure is presented in the crystal structure of the mouse mu opioid receptor. The helical secondary structure assigned with *Stride* [56] is shown as yellow rectangles. The gray residues in between TM5 and TM6 (MLSGSK) are absent in the crystal structure. The helical secondary structure of the wsMUR-TM model assigned with *Stride* is shown as blue lines. (B) Superposition of the mouse mu opioid receptor (yellow) and the wsMUR-TM model (blue). (C) Rendering from the “extracellular” viewpoint of the crystal structure of mouse mu opioid receptor, where the side chain of the mutated positions in wsMUR are depicted as blue spheres. The majority of mutations (50 out of 55) are located at the exterior of the structure. Five remaining positions (in green, see also green squares in Figure. 2A) are also rendered: Y130, T120, A306, N232, and K305. None of these positions are in direct contact with the irreversible antagonist β-FNA based on the crystal structure [15], where β-FNA was covalently attached to K235.

A comparative model of the human MUR transmembrane domain (288 residues, comprising sites 66–353) was obtained using known GPCR structures [25] (Figure 1). Based upon surface accessibility, 55 exterior transmembrane residues were selected for the computational redesign. As described in previous work, the calculations identify the probabilities of amino acids at variable positions among sequences that are expected to be water soluble by imposing overall energetic constraints such that (a) the amino acids are consistent with the remainder of the protein in terms of sterics, electrostatics and hydrogen-bonding (as guided by a molecular force field) and (b) a solvation or environmental energy is constrained to have a value expected for a globular protein of the same length, yielding exterior, hydrophilic residues expected for a water-soluble protein [4], [5]. A first calculation identified 31 of the targeted positions as having a strong preference for one amino acid, i.e., those sites where the probability one amino acid exceeded 0.8: A75E**^1.37^**, S78K**^1.40^**, I79K**^1.41^**, V83E**^1.45^**, F89K**^1.51^**, Y93E**^1.55^**, T120E**^2.54^**, K187K**^4.43^**, I188E**^4.44^**, V191E**^4.47^**, C192K**^4.48^**, A199E**^4.55^**, L202K**^4.58^**, M205E**^4.61^**, N232D**^5.36^**, L233K**^5.37^**, I240K**^5.44^**, F241K**^5.45^**, I244E**^5.48^**, M245E**^5.49^**, L248K**^5.52^**, V252E**^5.56^**, A289E**^6.42^**, V293K**^6.46^**, P297E**^6.50^**, I300K**^6.53^**, I303K**^6.56^**, I304E**^6.57^**, A306K**^6.59^**, L326K**^7.41^**, and V336K**^7.51^**. The superscript notation is consistent with the Ballesteros and Weinstein indexing system: (number of the transmembrane helix).(residue number relative to most conserved residue in transmembrane helix, which is assigned position 50) [34]. These mutations were introduced, and the corresponding residue identities were fixed in subsequent calculations. Similarly, second and third calculations respectively specified one (V82E**^1.44^**) and two (T72K**^1.34^** and L333E**^7.48^**) additional mutations. From the results of a fourth calculation, the most probable amino acid was selected at the remaining 21 positions, yielding a sequence and model structure for wsMUR-TM as presented in Figure 1. The designed sequence is presented in figure 2A.

The recent structure of the closely related murine MUR provides an opportunity to evaluate the structure and the location of the mutated positions in wsMUR-TM [15]. The human and mouse receptors have 94% sequence identity. The model of the human MUR and the murine crystal structure superimpose well (Figure 2B), particularly with regard to the transmembrane helices [35]. Only five positions in wsMUR-TM were not located in the exterior of the murine structure (T120E, Y130K, N232D, K305G, and A306K) and could in principle affect ligand binding (Figure 2C). In the murine structure, however, these five positions residues were not among the residues that directly contact beta-Funaltrexamine (β-FNA), an irreversible antagonist of the receptor [15].

### Overexpression, Purification, and Verification of wsMUR-TM

**Figure 3:**
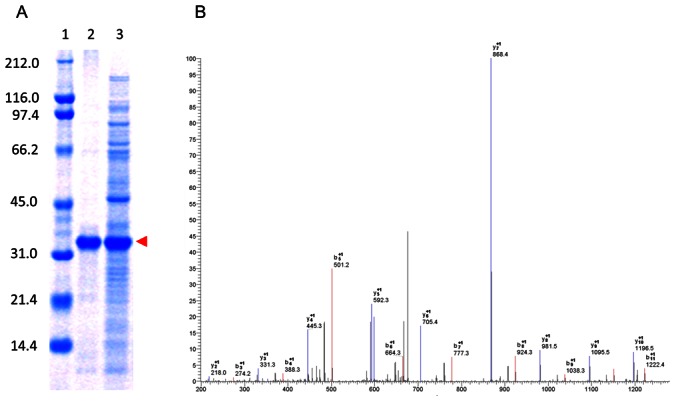
Overexpression and verification of wsMUR-TM. (A) A SDS-PAGE gel for wsMUR-TM is shown where lane 1 correspond to the standard, lane 2 to purified wsMUR-TM and lane 3 to expressed wsMUR-TM in the crude material. The band corresponding to the wsMUR-TM is indicated by a red arrow at 36 kDa. (B) Representative mass spectrometry data for fingerprinting of an identified peptide fragment is displayed (TATNIYIFNLAK; from the IC1-TM2 region).

Attempts to express the native full-length human MUR in *E. coli* were unsuccessful presumably due to the protein's toxicity. In contrast, wsMUR-TM expressed well and was isolated with high purity using affinity chromatography (Figure 3A). The yield was ∼20 mg/L of shake flask culture. An initial exposure to ∼0.1% sodium dodecyl sulfate (SDS) was required to purify the receptors. After dialysis to remove non-bound SDS, the purified variant was soluble at 6 mg/mL in buffer solution (130 mM NaCl, 20 mM NaHPO_4_, pH = 7.0). Using mass spectrometry (MS), fragments of the wsMUR were identified covering 37.6% of the designed sequences. One of the identified fragments of the receptor is presented in Figure 3B.

### Secondary Structure of wsMUR-TM

The secondary structure of the water-soluble variant was determined through circular dichroism (CD). The CD spectra indicated predominantly helical structures with a helical secondary structure content of ∼48% (estimations based on the molar ellipticity over the range 205 to 260 nm). The comparison of the helical content with that of the native human MUR expressed in yeast system in the presence of high concentration of detergent (0.1% SDS) is presented in table 1.

**Table 1:** Helical content comparison for the native and engineered receptors.

205–260 nm	wsMUR-TM (pH 7.0 in NaHPO_4_)	Native MUR (pH 7.0+0.1% SDS)
Helix	48.0%	40.6%
Turn	14.6%	18.9%
Others	37.4%	40.5%

wsMUR-TM: transmembrane-only water-soluble human mu receptor variant;

MUR: human mu receptor.

### Thermal Stability, Cholesterol Interaction, and Conserved Disulfide Bond

**Figure 4:**
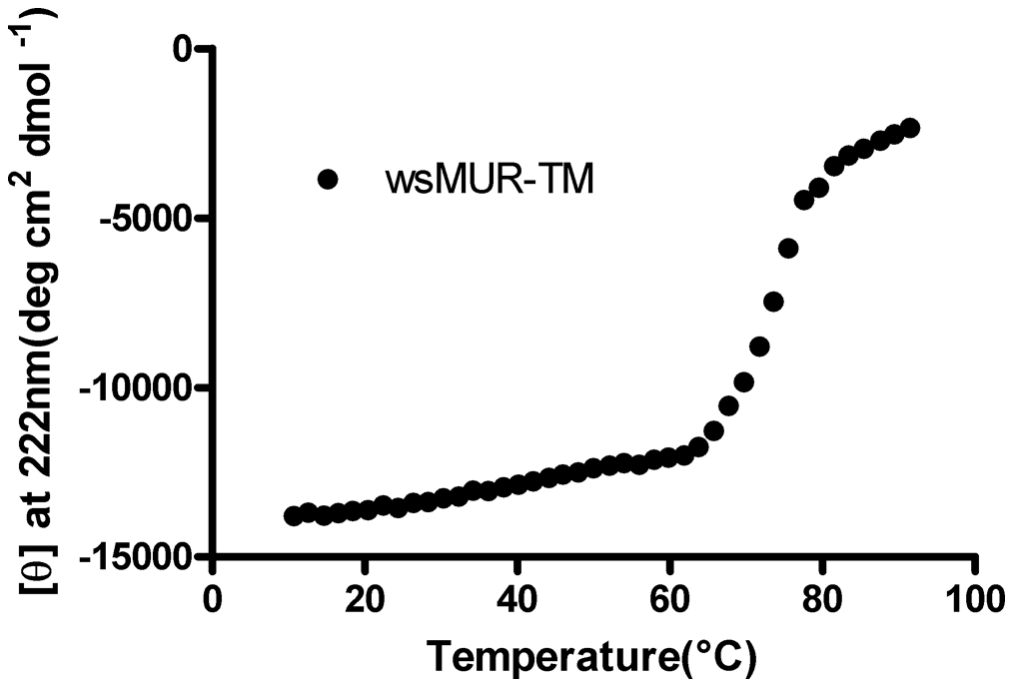
Mean residue ellipticity at 222 nm of wsMUR-TM in buffer solution (5 mM sodium phosphate, pH = 7.0) as a function of temperature, from 10 to 90°C. The spectrum of wsMUR-TM showed significant change near 62°C and an almost complete loss in molar ellipticity at 90°C.

**Figure 5:**
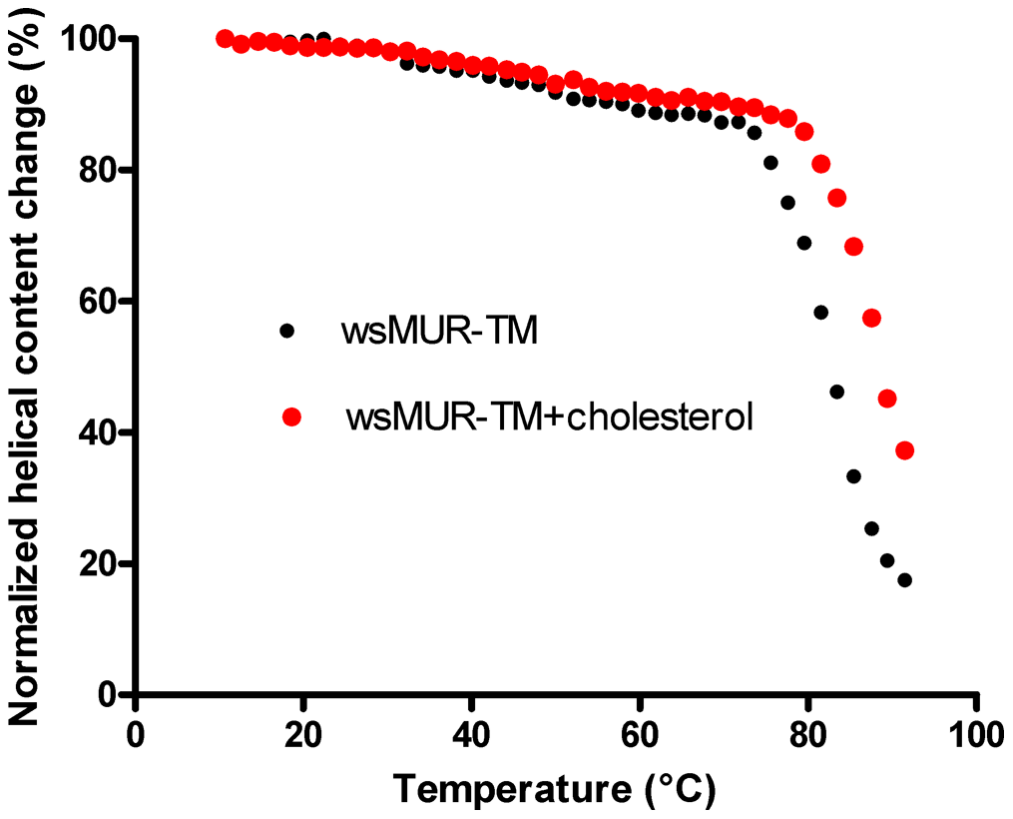
Molar circular dichroism (CD) derived percentage of the original helical content (determined at 222 nm) of wsMUR-TM in the absence (black dots) and the presence (red dots) of cholesterol in buffer solution (5 mM sodium phosphate, 0.01% SDS, pH = 7.0) as functions of the temperature. The addition of cholesterol stabilized the wsMUR-TM as indicated by the rightward shift of the thermostability curve.

As monitored by CD, wsMUR-TM started to lose ellipticity significantly near 62°C and was almost fully unfolded at 90°C (Figure 4). The stability of wsMUR-TM was also investigated upon addition of cholesterol, which has been found to modulate the stability of several GPCRs [14], . The inclusion of cholesterol caused a shift of the melting point from 82.9°C to 89.3°C, suggesting that it may stabilize the helical structure of wsMUR-TM (Figure 5) [14], [36].

CD and intrinsic tryptophan fluorescence were used to probe disulfide bond formation [37] in the water-soluble variant. The structure of wsMUR-TM was monitored with increasing concentrations of urea and the reducing agent 2-mercaptoethanol (2-ME). After addition of urea, the molar ellipticity at 222 nm and the intensity of the intrinsic tryptophan fluorescence of wsMUR-TM decreased. Even in 8 M urea, the protein retains some helical structure (Table 2). Upon addition of 2-ME, both the molar ellipticity and fluorescence further decreased, becoming more pronounced at the higher concentration of the reducing agent (200 mM). Thus the presence of an intramolecular disulfide bond is corroborated in the case of wsMUR-TM.

**Table 2:** Effects of denaturant and reducing agent on the wsMUR-TM.

	None	Urea(8 M)	Urea (8 M) 2-ME (25 mM)	Urea (8 M) 2-ME (200 mM)
Molar Ellipticity (%; 222 nm)	100.0	40.0	25.1	0.0
Fluorescence Peak Intensity (%; 300–350 nm)	100.0	28.4	23.9	4.5

wsMUR-TM: transmembrane-only water-soluble human mu receptor variant;

Values are normalized to the condition without denaturant or reducing agent (None). 2-ME: 2-mercaptoethanol.

### wsMUR Binding Assay

**Figure 6:**
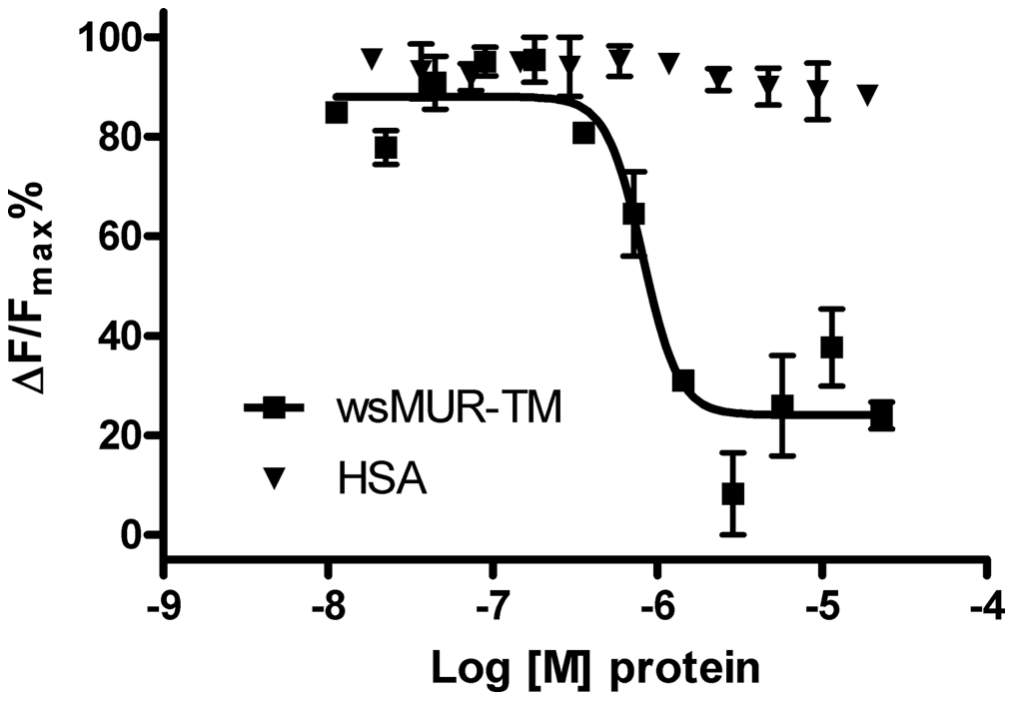
Binding competition assay between the human mu opioid receptor expressed in HEK293 cells and the µ opioid water-soluble variants. Inhibition of the native mu opioid receptor constitutive signal in the presence of increasing concentrations of wsMUR-FL (black dots, IC_50_ = 8.4×10^−7^ M, R^2^ = 0.9306) or wsMUR-TM (red squares, IC_50_ = 8.6×10^−7 ^M, R^2^ = 0.9067) in sodium phosphate buffer. Data for the negative control is also included, HSA (inverted green triangles). Data is used to calcualte HTRF ratios, and represent the mean ± standard error of mean of quadruplicates. ΔF is used for the comparison of different runs of the same assay which reflects the signal to background of the assay. ΔF = [(Ratio_sample_-Ratio_backgroud_)/Ratio_backgroud_](%).

Naltrexone binding was monitored using a competitive TR-FRET (time-resolved fluorescence resonance energy transfer) based assay with fluorescently labeled wild type MUR and a naltrexone-derived antagonist. The ratio of fluorescence emission at 665 nm and 620 nm decreased in a dose-dependent manner with increasing concentrations of wsMUR-TM. The determined *K*_d_ values for naltrexone were 65±1.8 nM (wsMUR-TM) (Figure 6). As a negative control, human serum albumin (HSA, a soluble helical protein), rather than a water-soluble variant, was introduced with no significant change in the fluorescence ratio upon HSA addition.

## Discussion

### Computational Design of wsMUR-TM

For many membrane proteins, the local environments of residues in the interior of the protein are similar to those observed in the interiors of globular proteins [38]–[41]. For such proteins, the exterior lipid-contacting residues, which are predominantly hydrophobic, could in principle be redesigned so as to yield a more water-soluble variant. This requires reliable structural information to determine which residues are on the exterior of the protein in the transmembrane region. Computational methods have been used to guide such redesign. The transmembrane domains of *Streptomyces lividans* (KcsA) [4], [42] and the α1 subunit of the nicotinic acetylcholine receptor (nAChR) from *Torpedo marmorata* [5] have been redesigned resulting in mutations to 30% and 18% of the respective proteins [4], [5], [43]. The water-soluble proteins retain structural and functional features of the parent membrane proteins. In these studies, experimentally derived structural information guided the selection of exterior residues and the computational redesign at these selected sites.

Herein, the computational redesign approach was extended to design functional water-soluble variant of a human GPCR without explicit *a priori* experimental structural information (when the effort was initiated). The GPCR system studied here is the largest protein yet targeted for solubilization via redesign (53 out of 288 residues, approximately 18% of the protein). The human MUR was selected due to its pharmacological relevance to understanding pain management and opioid addiction. wsMUR-TM was expressed in large quantities in a heterologous bacterial system and displayed structural and functional characteristics comparable with those of the native receptor.

The computationally guided redesign requires a structural model of the protein. Two aspects of the redesign process are expected to be sensitive to the accuracy of the model structure: the identification of exterior, transmembrane positions that are targeted for redesign and the amino acid probabilities used to determine the redesigned sequence. Given the similarity of known GPCR structures and the quality of the stuctures that can be obtained with comparative modeling, of these two aspects we expect the amino acid probabilities to be more sensitive to the detailed structural features of a given model structure. The recent structure of the closely related murine MUR provides an opportunity to evaluate the quality of the modeled structure and the location of the mutated positions in wsMUR-TM [15]. The human and mouse receptors have 94% sequence identity. The comparative model of the human MUR and the murine crystal structure superimpose well, particularly in the transmembrane region [35]. However, five targeted positions in wsMUR-TM were not located in the exterior of the murine structure (T120E, Y130K, N232D, K305G, and A306K). These five positions residues were not among the eleven that directly contact the ligand in the crystal structure; these residues compose the “binding site” and are those having an atom within 4 Å of the β-FNA ligand present in the crystal structure [15]. Thus, the binding properties of the wsMUR-TM are expected to be comparable with those of the native mu opioid receptor as demonstrated in this study. Although additional binding sites could potentially be introduced via computational redesign of the protein, we would not expect these to have affinities comparable to the wild type protein. In future work, details of the binding site of the wsMUR-TM can potentially be resolved using high-resolution structural approaches like NMR and x-ray crystallography.

### GPCR Expression in E. coli and Protein Purification

The expression of several GPCRs in *E. coli* has been achieved [44], but obtaining functional GPCRs in large quantities has generally been challenging. While functional human MUR has previously been successfully expressed in *E. coli* [45], the protein appeared as part of a fusion construct and was obtained at a low yield and remained unpurified. To our knowledge, successful induction of useful amounts of native MUR without a fusion partner (or with a His-tag) in *E. coli* has not been reported. The apparently toxic effects of the human MUR to the cells may explain the lack of such reports. Such toxicity was also observed in this study in attempts to express the native human MUR with a His-tag. However, production of His-tagged computationally designed human MUR variant (wsMUR-TM) was achieved. Thus, the toxicity of the native receptor appears to arise from hydrophobic residues located on the exterior surface of the receptor’s transmembrane region. The ability to express and purify large amounts of functional GPCRs from *E. coli* should greatly accelerate studies of the structure-function relationships for such receptors.

Since an initial exposure to 0.1% SDS was required during purification, the purified wsMUR-TM in solution may still contain small amounts of SDS due to the difficulty of removing SDS from proteins. In order to avoid protein aggregation, 0.01% of SDS was utilized in the final buffer solutions for functional assays. Using binding and crystallographic studies, we have shown that such small amounts of SDS do not disrupt the tertiary structure and/or the ligand binding capabilities of some proteins [46]. Conversely, much higher concentration of SDS (0.1%) and other anionic detergents are required for the “solubilization” of the native human MUR [47].

### Protein Structure Characterization and Thermostability

Consistent with the secondary structure of the native human MUR expressed in yeast [48], [49], the CD spectra of the wsMUR-TM displayed a predominantly helical structure (48%) which is comparable with the native full length MUR [48]. Lower percentage of the helical content in the native full length MUR in the literature may be due to the inclusion of the N and C terminus and the higher concentration of the detergent (0.1% SDS).

Intrinsic tryptophan fluorescence was used to provide qualitative information of the conformations adopted by the water-soluble receptors; wsMUR-TM contains just six tryptophan residues (W135**^2.69^**, W194**^4.50^**, W228**^EC2^**, W230**^EC2^**, W295**^6.48^**, and W320**^7.35^**). Of particular interest are the tryptophan residues located in the partially buried transmembrane locations of the model structure (underlined above). The fluorescence associated with these residues is expected to be sensitive to the local hydrophobic environment and overall folding of the protein. The observed decrease in the tryptophan fluorescence and the red shift in the emission with increasing denaturant (urea) concentration suggest that at least some of these tryptophan residues are located in the interior of the protein.

The decrease of the tryptophan fluorescence under denaturing conditions and in the presence of 2-ME is consistent with the changes in CD spectra observed under similar conditions. The requirement of the reducing agent to fully denature and unfold the protein indicates the relevance of an intramolecular disulfide bond in stabilizing the receptor structure. Although these observations suggest the presence of a disulfide bond, they do not specify which bond is formed given the existence of 11 cysteine residues in wsMUR-TM. However, the CD and ligand-binding studies are consistent with the adoption of the proper protein tertiary structure and by extension with the formation of the correct disulfide bond.

With the exception of rhodopsin, GPCRs are not generally stable and this represents one of the major obstacles for structural studies [10]. Many strategies have been employed to overcome this problem, such as the use of stabilizing ligands [50], stabilizing mutations [51], and high salt concentrations in solution [13]. In the present study, wsMUR-TM that is both soluble in aqueous media and thermally stable was successfully generated by redesigning the protein. The thermostability of wsMUR-TM improved significantly in the presence of cholesterol. These results are consistent with recent observations that the introduction of cholesterol hemisuccinate increases the thermostability of the A_2A_ adenosine receptor in detergent micelles [14]. Studies have demonstrated that interactions between cholesterol and GPCRs can play an important role in modulating the structural stability as well as the function of the receptors [36], [52]. Moreover, cholesterol is present in the recently solved crystal structure of the murine mu opioid receptor [15]. Two mechanisms have been proposed regarding cholesterol and membrane proteins: *a*) direct interaction between the receptor and the cholesterol molecule [36] and/or *b*) a modification of the membrane microenvironment of the protein by cholesterol [53]. Given that the experiments here were performed in aqueous solution, the first mechanism appears to apply to the interactions with wsMUR-TM. One of the disadvantages of designing a water-soluble variant of an integral protein is the inability to study the mechanism of the membrane microenvironment modification given the absence of a membrane. Despite this limitation, it is noteworthy that the mutations of the lipid-contacting positions do not preclude the well-known interactions that exist between the native receptor and relevant molecules such as cholesterol. The finding that the wsMUR-TM thermostability increased in the presence of cholesterol together with the previous finding that the water-soluble α1 subunit of the nicotinic acetylcholine receptor (nAChR) recovers the protein-lipid interactions of the native receptor [5], [54], supports the power and flexibility of the solubilization approach in maintaining similar interactions as those seen in the native membrane-soluble protein.

### Ligand Binding Properties of the wsMUR-TM

A recently developed methodology which uses a fluorescently labeled ligand and the native MUR [55] was used to investigate the ligand-binding capabilities of the water-soluble receptors. This binding assay has been applied to study several GPCRs and particularly to MUR, where the *K*_i_ values for the morphinan opioids naloxone and naltrindole were estimated (5.1 nM and 8.1 nM for naloxone and naltrindole, respectively) and found to be in agreement with values obtained using other techniques [55], wsMUR-TM competes with native MUR expressed in HEK293 cells for the potent opioid antagonist naltrexone. This study clearly demonstrates that the wsMUR-TM can compete with the native MUR for the fluorescent antagonist with binding affinities in nM range. The HSA (negative control) results indicate that the interaction of the water-soluble variant with naltrexone is selective and specific.

### Conclusions and Future Directions

The findings reported in this study open new tools for the characterization of GPCRs and the human MUR. The computational design approach could be applied more broadly to other receptors in the GPCR superfamily, particularly those whose structure may not be known *a priori,* via the use of comparative (homology) modeling. The wsMUR-TM over-expressed in the *E. coli* system would be amenable for NMR spectroscopy and other structural studies in the presence and absence of opioid ligands. The water solubility of the receptor and its high yield in a bacterial production system offer great advantages for further pharmacological characterization as well as drug refinement and discovery.
